# The Impact of Optical Coherence Tomography in the Early Identification of Children with Sickle Cell Retinopathy

**DOI:** 10.1155/2022/9131423

**Published:** 2022-08-29

**Authors:** Ashraf Abdelkader, Mohamed Shaaban, Mahmoud M. Zahran, Mostafa F. Mohammed, Anas M. Ebrahim, Ahmed I. Galhoom

**Affiliations:** ^1^Department of Pediatrics, Faculty of Medicine for Boys, Al-Azhar University, Cairo, Egypt; ^2^Scientific Research and Continuous Medical Education Unit, Al Ansari Specialist Hospital, Yanbu, Saudi Arabia; ^3^Department of Ophthalmology, Faculty of Medicine for Boys, Al-Azhar University, Cairo, Egypt

## Abstract

**Introduction:**

Sickle cell disease is characterized by the production of abnormal hemoglobin, which affects hemoglobin molecule stability during hypoxia and leads to the formation of sickle cells, resulting in increased hematic viscosity, hemolysis, and microvascular blockage. Vasoocclusion is assumed to be the primary cause of vision-threatening retinopathy in sickle cell disease. The aim of this study was to improve the early detection of sickle cell retinopathy (SCR) in children with sickle cell disease (SCD) and investigate the link between systemic and ocular symptoms.

**Methods:**

This cross-sectional study comprised children with SCD. The patient files provided a detailed medical history. The laboratory tests included a blood count, reticulocyte count, and Hb electrophoresis. The slit lamp, fundus, optical coherence tomography (OCT), and optical coherence tomography angiography (OCTA) were all part of the ophthalmological examination.

**Results:**

The study comprised 15 children with sickle cell disease who met the inclusion and exclusion criteria, with a mean age of 11.15 ± 1.29 years. Nine of the children were males (60%) and six were females (40%). 8 (53.3%) of the children had Hb SS, three (20%) had Hb SC, three (20%) had Hb SB^+^, and one (6.7%) had Hb SB^0^. Four children (26.7%) had poor visual acuity. A fundus examination revealed significant abnormal findings in 12 of the 7 children's eyes (40 percent). Macular thinning was detected by OCT in 10 eyes of 7 children (33.3%). Flow voids at the deep retinal capillary plexus were detected by OCTA in 10 eyes of 7 children (33.3%). Longer disease duration, higher reticulocytic percent, more painful crises, and noncompliance with hydroxyurea medication were all linked to the existence of eye abnormalities on fundus examination and OCT.

**Conclusion:**

OCTA can show early retinal damage in sickle cell patients with macular changes. Sickle cell retinopathy is usually associated with more severe disease.

## 1. Introduction

Sickle cell disease (SCD) is a chronic hemolytic illness characterized by the generation of aberrant hemoglobin, which affects hemoglobin (Hb) molecule stability during hypoxemia with the stacking of abnormal Hb onto monofilaments and ultimately forms unique sickle cells, resulting in increased hematic viscosity, hemolysis, and microvascular blockage [1]. It is an autosomal recessive condition caused by abnormalities in the hemoglobin B-chain. The most common genotypes of sickle cell disease include homozygotes Hb SS and heterozygotes that may be Hb SC, HbSB^0^, or Hb SB^+^ [2]. Patients with Hb S*β*-thalassemia^0^ have a clinical phenotype similar to those with Hb SS, and both patients with Hb SS and HbSB^0^ do not have any adult Hb [3]. Microvascular occlusion and hemolytic anemia produce vasculopathy, inflammation, and progressive ischemic organ damage, resulting in a variety of systemic consequences, including stroke, pulmonary hypertension, splenic dysfunction, renal failure, liver dysfunction, and leg ulcers [4]. Painful crises constitute the most distinguishing clinical feature of SCD; some patients have as many as 6 or more episodes annually, whereas others may have an episode only at great intervals or none, with each patient having a consistent pattern of frequency [5].

Sickle cell disease has a variety of ocular symptoms, including conjunctival comma, hyphema, iris atrophy, and cataract [6]. The most common and severe ocular manifestation of this illness, sickle cell retinopathy (SCR), poses the greatest risk of visual impairment. It is caused by retinal ischemia produced by erythrocytes sickling in the arterioles, resulting in vascular blockage in the peripheral retina. Sickle cell retinopathy is classified as either nonproliferative or proliferative. Salmon patch hemorrhage, black sunburst, iridescent patches, and increased vascular tortuosity are all features of nonproliferative retinopathy. Goldberg's stages of proliferative retinopathy are as follows: (1) peripheral arteriolar blockage, (2) arteriovenous anastomosis at the retinal border, (3) peripheral neovascularization with sea fan, (4) vitreous hemorrhage, and (5) retinal detachment [7, 8]. Early signs of sickle cell retinopathy exist in children, albeit the frequency and age of start vary among research [9].

Sickle cell maculopathy (SCM), a localized retinal thinning typically found temporally to the macula, is a new consequence. SCM is not usually clinically visible by indirect ophthalmoscopy, and when it is, it presents as a marginally changed reflex. SCM is easily identified using spectral domain optical coherence tomography (SD-OCT) and optical coherence tomography angiography (OCTA). It is distinguished by disruption of the inner retinal layers or both the inner and intermediate retinal layers as a result of vascular obstruction [10]. In a single research study, SCM was discovered in a teenage population over the age of ten [11]. Patients with SCM have no visual signs. However, at microperimetry testing in adults, retinal sensitivity in areas of macular thinning is diminished [12].

Identifying sickle cell maculopathy and retinopathy in a pediatric SCD population is the major goal of this investigation. Moreover, this study aims to examine the link between systemic characteristics and ocular signs in children and adolescents with SCD to gain further insight into the condition.

## 2. Patients and Methods

This cross-sectional study was performed according to the STROBE Checklist and comprised 15 patients with SCD who were randomly selected from the Pediatric Clinic at Al-Ansari Hospital in Yanbu, KSA, between July 2019 and March 2021. Before participating, each patient's guardian provided informed consent. The protocols used in this investigation were authorized by the institutional Scientific Research Ethical Committee (approval: 19-7/2) and were in compliance with the Declaration of Helsinki.

Individuals with congenital or chronic ocular disorders, chronic hemolytic anemia (excluding sickle cell), diabetes mellitus, or any other chronic medical illness that may affect the eye were excluded.

The data collected from the patients' files included the age at disease diagnosis, disease duration, transfusion history including type (simple/exchange) and indication, hydroxyurea therapy, and compliance with the therapy was assessed using the report of dose taking obtained by the patient's parents and emphasized by checking the prescription refill and pill count; a cutoff point of less than 80% was considered poor compliance. A history of painful crises [frequent 6 times or more annually [5] or not frequent], avascular necrosis of the bones, acute chest syndrome, ocular symptom, cerebral stroke (occult or overt), and initial Hb pattern (SS, SC, SB^+^, SB^0^) at time of diagnosis was taken.

### 2.1. Laboratory Examination

Sysmex XT-1800i (Sysmex, Kobe, Japan) was utilized to assess total blood count, reticulocyte count was manually calculated using Brilliant cresyl blue stain, and Hb electrophoresis by high-performance liquid chromatography utilizing Helena V8 completely automated capillary electrophoresis equipment (Helena Biosciences Europe, UK).

### 2.2. Ophthalmological Examination

Experienced ophthalmologists performed slit lamp, fundus examination, and visual acuity tests on all patients. SOLO-OCT of the macular region was performed with a total of 25 scans spaced by 237 microns. A fixation target was utilized to center the scan in the fovea. In addition, all scans were analyzed for profile irregularities associated with retinal thinning. OCTA (RS-3000 OCT Retina Scan Advance 2, Nidek Co., Japan). Scans were centered on the fovea and had a field of view of 3 × 3 mm and 6 × 6 mm. A scan was performed above the retinal thinning with a 3 mm field of view on each patient with SCM. All photos with a high level of artifacts were excluded.

### 2.3. Statistical Analysis

Statistical analysis was carried out using the SPSS computer package version 25.0 (IBM SPSS, Armonk, NY; IBM Corp., USA). For descriptive statistics, the mean ± SD was used for quantitative variables while the number and percentage were used for qualitative variables. In analytic statistics, Chi-squared test was used to assess the differences in frequency of qualitative variables, while one-way ANOVA test was applied to assess differences in means of quantitative variables between Hb genotypes and independent samples *t*-test to assess differences in means of quantitative variables according to eye abnormalities. Statistical methods were verified, assuming a significant level of *p* < 0.05 and a highly significant level of *p* < 0.001.

## 3. Results

The study included 15 children diagnosed with sickle cell disease, fulfilling the inclusion and exclusion criteria, with a mean age of 11.15 ± 1.29 years, ranging from 8 to 14 years; 9 children were males (60%) and 6 were females (40%). Their mean age at diagnosis was 22.7 ± 9.07 months, ranging from 9 to 38 months, and the mean duration of illness was 9.26 ± 1.4 years, ranging from 7 to 11.5 years. History of blood transfusion showed simple transfusion in 12 children (80%) and exchange transfusion in 3 children, one (6.7%) with suspected stroke and two (13.3%) with acute chest syndrome. Before the study, their mean Hb level was 8.13 ± 1.01 gm/dL ranged from 6.9 to 10 gm/dL, their mean Hb F % was 8.4 ± 3.66 ranged from 4 to 18%, and their mean retics % was 9.3 ± 2.4 ranged from 4 to 13%. One child (6.7%) experienced seizures with suspected silent stroke, 5 children (33.3%) suffered from frequent headaches, and 4 children (26.7%) suffered from decreased visual acuity. Painful crisis occurred frequently in 6 children (40%) and infrequently in other 6 children. Hydroxyurea therapy was prescribed for 12 children, of which 7 children (46.7%) were compliant with the therapy and 5 children (33.3%) were not compliant. Fundus examination showed abnormal findings in 12 eyes of 7 children (40%), where both eyes of 3 children and one eye of a child showed tortuosity, salmon patches, and black sunspots; both eyes of one child and one eye of another child showed tortuosity and salmon patches, and both eyes of a child showed only tortuosity. By OCT, macular thinning (atrophy) was detected in 10 eyes of 7 children (33.3%). By OCTA, flow voids at the deep retinal capillary plexus were detected in 10 eyes of 7 children (33.3%) and flow voids at the superficial retinal capillary plexus were detected in 4 eyes of 3 children (13.3%) (Table 1).

**Figure 1:**
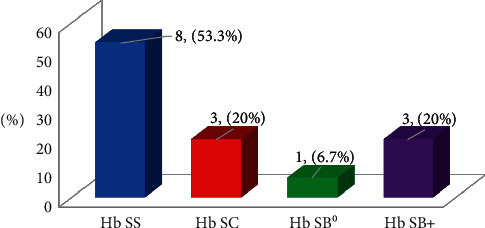
Distribution of Hb genotype among children with sickle cell disease.

Their Hb genotype by electrophoresis revealed 8 children (53.3%) with Hb SS, 3 (20%) with Hb SC, 3 (20%) with Hb SB^+^, and 1 child (6.7%) with Hb SB^0^ (Figure 1).

**Figure 2:**
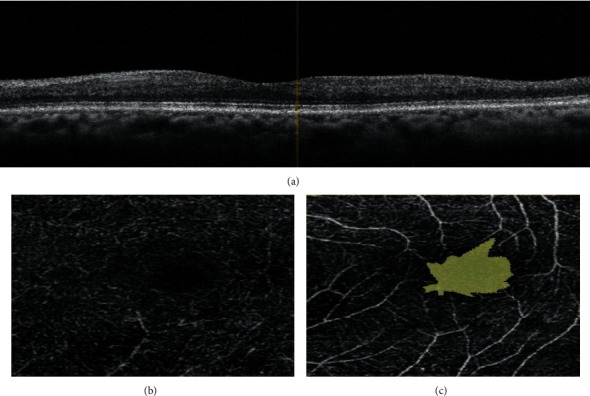
13-year-old boy with Hb SS showing (a) macular thinning by OCT, affection of deep (b) and superficial (c) vascular plexuses by OCTA.

No significant differences were found between different Hb genotypes regarding age, gender, and age at diagnosis. However, in children with Hb SS and Hb SB^0^, the mean duration of illness and reticulocytic percent prior to the study were significantly higher, and the mean Hb level and Hb F % prior to the study were significantly lower than other Hb genotypes. Significantly, a history of simple blood transfusion was evident in all Hb genotypes except Hb SB^+^. Two children with Hb SS and the child with Hb SB^0^ had a history of exchange transfusion. Seizures were only experienced by a child (12.5%) with Hb SS, frequent headache was insignificantly experienced by 3 children (37.5%) with Hb SS, a child (33.3%) with Hb SC and the child with Hb SB^0^, and decreased visual acuity was insignificantly experienced by 2 children (25%) with Hb SS, a child (33.3%) with Hb SC and the child with Hb SB^0^. Frequent painful crisis was significantly evident in 4 children (50%) with Hb SS, a child (33.3%) with Hb SC, and a child with Hb SB^0^. Significantly, treatment with hydroxyurea was not compliant by 3 children (37.5%) with Hb SS, a child (33.3%) with Hb SC, and a child with Hb SB^0^. For each child, a fundus examination was done separately for both eyes. Abnormal findings were significantly higher among children with Hb SS (10 out of 16 eyes; 62.5%, bilateral in 5 children), in the child with Hb SB^0^ (one of both eyes; 50%), then among a child with Hb SC (1 out of 6 eyes; 16.7%) while no abnormalities were detected in children with Hb SB^+^. By OCT, macular thinning (atrophy) was found significantly higher among children with Hb SS (9 out of 16 eyes; 56.3%, bilateral in 3 children and unilateral in 3 children) (Figure 2) and a child with Hb SC (unilateral in one eye; 16.7%) while no abnormalities were detected in children with Hb SB^0^ and Hb SB^+^. By OCTA, flow voids at the deep retinal capillary plexus (OCTA) were found significantly higher among children with Hb SS (9 out of 16 eyes; 56.3%, bilateral in 3 children and unilateral in 3 children) (Figure 2) and a child with Hb SC (unilateral in one eye; 16.7%) while no abnormalities were detected in children with Hb SB^0^ and Hb SB ^+^ whereas flow voids at the superficial retinal capillary plexus were found in 2 eyes (12.5%) of a child with Hb SS figure 2, one eye (16.7%) in a child with Hb SC, one eye (50%) in a child with Hb SB^0^ while no changes were detected in children with Hb SB^+^ (Table 2).

According to the Hb genotype and ocular manifestations, this study included eight Hb SS patients. One of them exhibited bilateral abnormal fundus examinations, macular atrophy on OCT, and flow voids in the deep and superficial retinal plexus on OCTA. Two cases had bilateral abnormal fundus examination, bilateral macular atrophy by OCT, and bilateral flow void at deep retinal plexus by OCTA. Two patients had abnormal bilateral fundus examinations; OCT revealed unilateral macular atrophy; and OCTA revealed unilateral flow void at the deep retinal plexus. One patient had unilateral macular atrophy on OCT and a unilateral flow void at the deep retinal plexus on OCTA, whereas the other two patients had normal fundus, OCT, and OCTA examinations. In addition, only one of the three Hb SC patients displayed unilateral fundus abnormalities, unilateral macular atrophy by OCT, and unilateral flow void at the deep and superficial retinal plexus by OCTA, while the only patient with Hb SB^0^ exhibited unilateral abnormal fundus examination and unilateral flow void at the superficial retinal plexus by OCTA. Finally, three patients with Hb SB^+^ had normal eye examinations by fundus, OCT, and OCTA (Table 3).

Presence of eye abnormalities by fundus examination was significantly associated with longer disease duration, lower Hb F %, increased retics %, more frequent painful crisis, and noncompliant with hydroxyurea therapy. Although a history of repeated exchange transfusion occurred only in children with eye abnormalities, there was no significant difference (Table 4).

Presence of macular thinning (atrophy) by OCT and flow voids at the deep retinal capillary plexus by OCTA were significantly associated with longer disease duration, increased retics %, more frequent painful crisis, and noncompliant with hydroxyurea therapy. The mean Hb F % was lower in children with macular thinning but with no significant difference. Though history of repeated exchange transfusion occurred only in children with eye abnormalities, there was no significant difference (Table 5).

## 4. Discussion

SCD is a severe chronic hemolytic condition that causes hemolysis, vascular damage, and tissue ischemia in various organs [13]. SCD pathophysiology is based on hemoglobin S polymerization, which causes hemodynamic anomalies, such as sludging and arterial blockage in the microvasculature, including the retina [14]. Vasoocclusive retinal disease can be proliferative or nonproliferative [15].

The primary purpose of this study is to detect sickle cell maculopathy and retinopathy in children with SCD. In addition, this study intends to investigate the relationship between systemic features and ocular symptoms in children with SCD to acquire a better understanding of the condition.

In terms of age and gender distribution, no significant disparities were found between groups, ensuring that our findings are consistent across all groups.

Although according to our results, the decreased visual acuity was insignificantly experienced by 2 children with Hb SS (25%), a child with Hb SC (33.3%), and the only child with Hb SB^0^, the ophthalmoscopic examination revealed significant nonproliferative retinopathy in 12 eyes of 7 children (40%). Nonproliferative retinopathy symptoms were reported in HbSS, HbSB^0^, and HbSC patients. None were discovered in HbSB^+^ genotypes. Gill and Lam [16] found that HbSC children had earlier ocular signs than HbSS and HbSB^+^ children and so recommended that this genotype be screened at a younger age. The development of SCR may be associated with a longer life expectancy and ongoing retinal ischemia, which stimulates VEGF production, and total capillary blockage [17]. Early symptoms of retinopathy in children with HbSS have been documented by Ronsberg and Hutcheson [18], Eruchalu et al. [19], and Tantawy et al. [20] particularly in the presence of severe systemic problems.

In our investigation, OCT and OCTA detected SCM as macular thinning and affection of flow voids in the deep vascular plexus, respectively, in 10 eyes of 7 patients, 9 eyes with Hb SS children, and 1 eye with a child with Hb SC. In agreement with Grego et al. [21], early prediction of SCR and SCM was detected in children by SD-OCT and OCTA. These two retinal problems were more prevalent in children with a history of hemolysis. Leitão Guerra et al. [22] detected a relationship between SCM and age and found SCM affects just 20% of the population under the age of 18, but 100% of the older population. Although sickle cell maculopathy may be detected by indirect slit-lamp ophthalmoscopy and retinal fluorescein angiography, OCT has greater diagnosis accuracy. OCTA enables the evaluation of deep and superficial vascular plexuses individually, which is not achievable with traditional fluorescein angiography [22]. Because terminal arteries along the horizontal meridian are more prone to occlusion in patients with this condition, retinal thinning regions are more frequently found temporally to the macula [23,24]. OCTA of thinned macular areas reveals flow void affections in the superficial and deep vascular plexuses in both children and adults, albeit the areas of flow void affection are more widespread in the deep arteries due to greater oxygen demand [24, 25]. In our study, the flow void significantly involved the deep vascular plexus, with insignificant affection of the superficial plexus in agreement with Grego et al. [21] who concluded that sickle cell maculopathy can progress, first affecting the flow voids in the deep vascular plexus and then the superficial plexus.

In our study, we found the presence of eye abnormalities by fundus examination was significantly associated with longer disease duration, lower HbF %, increased retics %, history of repeated exchange transfusion, more frequent painful crises, and poor compliance with hydroxyurea therapy, in agreement with Fadugbagbe et al. [15]. Conversely, Fox et al. found a higher correlation between elevated hemoglobin levels and retinopathy in subjects with sickle cell anemia [26]. Lower hemoglobin levels are linked to higher hemolysis rates and they are often found in patients with more severe systemic complications [27]. Furthermore, hemolysis releases hemoglobin into plasma and causes the production of reactive oxygen species, which reduce the concentration of nitric oxide. The latter regulates the basal tone of vessel dilation and inhibits platelet aggregation and activation of hemostasis [2]. Thus, nitric oxide reduction causes vascular endothelial damage and alteration of platelet aggregation, with alteration of peripheral retinal perfusion and possible development of sickle cell retinopathy.

Our OCT findings revealed the presence of macular thinning was significantly associated with longer disease duration, increased reticulocytic percent, history of repeated exchange transfusion, more frequent painful crisis, and noncompliance with hydroxyurea therapy. Few studies correlate sickle cell maculopathy with systemic indices and manifestations, in agreement with Grego et al. [21]. Also, Dell' Arti et al. [10] showed a correlation between retinal thinning and irregularities in the intake of hydroxyurea, lower percentages of fetal hemoglobin, and nonregular transfusions.

Furthermore, we discovered a high incidence of systemic and ocular manifestations in Hb SS patients, who also reported higher noncompliance with hydroxyurea therapy than patients with other Hb genotypes, an association that warrants further investigation. So, we recommend studying a larger sample size of compliant patients only to check if the incidence of systemic and ocular manifestations remains high in spite of compliance to therapy or not.

### 4.1. Limitations

The small sample size is a limitation that may have an impact on the validity of the OCT's utility in determining its effectiveness. Because there were so many variables to consider, a larger sample size would have been more convenient.

## 5. Conclusion

It is possible to visualize early retinal affection in sickle cell patients with macular alterations using OCTA. Because sickle cell retinopathy is one of the manifestations of sickle cell disease, it is usually associated with more severe disease among affected children.

## Figures and Tables

**Table 1:** General, clinical, laboratory, and ocular investigations among the studied children.

Variables		*N* = 15 (%)
Age (years)	Mean ± SD Min—Max	11.15 ± 1.29
		8–14
Age at diagnosis (months)	Mean ± SD Min—Max	22.7 ± 9.07
		9–38
Disease duration (years)	Mean ± SD Min—Max	9.26 ± 1.4
		7–11.5
Gender	Male	9 (60.0)
	Female	6 (40.0)
Simple blood transfusion	Present	12 (80.0)
Exchange transfusion	One time (suspected stroke)	1 (6.7)
	One time (acute chest syndrome)	2 (13.3)
Hb (gm/dL) prior to study	Mean ± SD Min—Max	8.13 ± 1.01
		6.9–10
Hb F (%) prior to study	Mean ± SD Min—Max	8.4 ± 3.66
		4–18
Retics (%) prior to study	Mean ± SD Min—Max	9.3 ± 2.4
		4–13
Neurological symptoms	Seizures (silent stroke)	1 (6.7)
	Frequent headache	5 (33.3)
Ocular symptoms	Decreased visual acuity	4 (26.7)
Painful crisis	Frequent	6 (40.0)
	Infrequent	6 (40.0)
Hydroxyurea therapy	Not compliant	5 (33.3)
	Compliant	7 (46.7)
Fundus exam (*n* = 30)	Abnormal findings^1^	12 (40.0)
OCT (*n =* 30)	Macular thinning^2^	10 (33.3)
OCTA (*n =* 30)	Flow voids at the deep retinal capillary plexus^3^	10 (33.3)
	Flow voids at the superficial retinal capillary plexus^4^	4 (13.3)

OCT: optical coherence tomography, OCTA: optical coherence tomography angiography. ^**1**^From a total of 30 eyes of 15 children, abnormal findings were detected in 12 eyes of 7 children. ^**2**^From a total of 30 eyes of 15 children, macular thinning was detected in 10 eyes of 7 children.^**3**^From a total of 30 eyes of 15 children, flow voids at the deep retinal capillary plexus were detected in 10 eyes of 7 children. ^**4**^From a total of 30 eyes of 15 children, flow voids at the superficial retinal capillary plexus were detected in 4 eyes of 3 children.

**Table 2:** Relation of different Hb genotypes and study variables.

Variables		Hb SS *n* = 8 (%)	Hb SC *n* = 3 (%)	Hb SB^0^*n* = 1 (%)	Hb SB + *n* = 3 (%)	*P*-value
Age (years)		11.7 ± 1.3	11.3 ± 0.3	11.0	9.5 ± 0.3	0.064
Age at diagnosis (months)		21.0 ± 9.8	24.7 ± 13.0	18.0	26.7 ± 4.2	0.780
Disease duration (years)		10.0 ± 1.2	9.2 ± 0.9	9.5	7.3 ± 0.6	0.027^*∗*^

Gender	Male	5 (62.5)	2 (66.7)	0 (0.0)	2 (66.7)	0.652
	Female	3 (37.5)	1 (33.3)	1 (100.0)	1 (33.3)	

Hb prior to study		7.5 ± 0.4	8.5 ± 0.1	6.9	9.8 ± 0.3	<0.001^*∗*^
Hb F (%) prior to study		6.4 ± 0.5	8.0 ± 1.0	6.0	15.0 ± 2.6	<0.001^*∗*^
Retics (%) prior to study		10.9 ± 1.5	8.7 ± 0.6	9.5	5.7 ± 1.5	0.001^*∗*^

Simple blood transfusion	Present	8 (100.0)	3 (100.0)	1 (100.0)	0 (0.0)	0.002^*∗*^

Exchange transfusion	One time (suspected stroke)	1 (12.5)	0 (0.0)	0 (0.0)	0 (0.0)	0.208
	One time (acute chest syndrome)	1 (12.5)	0 (0.0)	1 (100.0)	0 (0.0)	

Neurological symptoms	Seizures (silent stroke)	1 (12.5)	0 (0.0)	0 (0.0)	0 (0.0)	0.569
	Frequent headache	3 (37.5)	1 (33.3)	1 (100.0)	0 (0.0)	
Ocular symptoms	Decreased visual acuity	2 (25.0)	1 (33.3)	1 (100.0)	0 (0.0)	0.270

Painful crisis	Frequent	4 (50.0)	1 (33.3)	1 (100.0)	0 (0.0)	0.011^*∗*^
	Infrequent	4 (50.0)	2 (66.7)	0 (0.0)	0 (0.0)	

Hydroxyurea therapy	Not compliant	3 (37.5)	1 (33.3)	1 (100.0)	0 (0.0)	0.010^*∗*^
	Compliant	5 (62.5)	2 (66.7)	0 (0.0)	0 (0.0)	

		Hb SS *n* = 16 (%)	Hb SC *n* = 6 (%)	Hb SB^0^*n* = 2 (%)	Hb SB + *n* = 6 (%)	*P*-value
Fundus exam	Abnormal findings^**1**^	10 (62.5)	1 (16.7)	1 (50.0)	0 (0.0)	0.032^*∗*^
OCT	Macular thinning	9 (56.3)	1 (16.7)	0 (0.0)	0 (0.0)	0.036^*∗*^
OCTA	Flow void at the deep retinal capillary plexus	9 (56.3)	1 (16.7)	0 (0.0)	0 (0.0)	0.036^*∗*^
	Flow void at the superficial retinal capillary plexus	2 (12.5)	1 (16.7)	1 (50.0)	0 (0.0)	0.345

^*∗*^Significant.^**1**^Children with Hb SS showed tortuosity, salmon patches, and black sunspots in 3 children (6 eyes), tortuosity and salmon patches in one child (2 eyes), and another one child (2 eyes) showed tortuosity only. A child with Hb SC showed tortuosity, salmon patches, and black sunspots in one eye and the child with Hb SB^0^ showed tortuosity, salmon patches, and black sunspots in one eye.

**Table 3:** The ocular findings and hemoglobin genotypes among the studied patients.

HB genotype(no)		Fundus examination	OCT	OCTA	
				Deep retinal capillary plexus	Superficial retinal capillary plexus
Hb SS (8)	2 patients	Normal	Normal	Normal	Normal
	1 patient	Bilaterally abnormal	Bilateral macular atrophy	Bilateral flow void	Bilateral flow void
	2 patients	Bilaterally abnormal	Bilateral macular atrophy	Bilateral flow void	Normal
	2 patients	Bilaterally abnormal	Unilateral macular atrophy	Unilateral flow void	Normal
	1 patient	Normal	Unilateral macular atrophy	Unilateral flow void	Normal
Hb SC (3)	1 patient	Unilaterally abnormal	Unilateral macular atrophy	Unilateral macular atrophy	Unilateral flow void
	2 patients	Normal	Normal	Normal	Normal
Hb SB^+^ (3)	3 patients	Normal	Normal	Normal	Normal
Hb SB^0^ (1)	1 patient	Unilaterally abnormal	Normal	Normal	Unilateral flow void

**Table 4:** Relation between eye abnormalities by fundus examination and different study variables.

Variables		Eye abnormalities *n* = 12 (%) (7 children)	No eye abnormalities *n* = 18 (%) (8 children)	*P*-value
Disease duration (years)		10.0 ± 1.0	8.7 ± 1.4	0.009^*∗*^
Hb F (%) prior to study		6.58 ± 0.9	9.61 ± 4.2	0.021^*∗*^
Retics (%) prior to study		10.37 ± 1.4	8.58 ± 2.6	0.039^*∗*^

Exchange transfusion ^1^	One time (suspected stroke)	1/7 (14.3)	0/8 (0.0)	0.117
	One time (acute chest syndrome)	2/7 (28.6)	0/8 (0.0)	

Painful crisis	Frequent	8 (66.7)	4 (22.2)	0.020^*∗*^
	Infrequent	4 (33.3)	8 (44.4)	

Hydroxyurea therapy ^2^	Not compliant	5/7 (71.4)	0/8 (0.0)	0.010^*∗*^
	Compliant	2/7 (28.6)	5/8 (62.5)	

^1^Occurred in 3 children, all with eye abnormalities by fundus examination. ^2^Prescribed in 12 children, 7 with eye abnormalities and 5 without abnormalities by fundus examination.

**Table 5:** Relation between eye abnormalities by OCT and OCTA and different study variables.

Variables		Eye abnormalities (macular thinning and flow void at the deep retinal capillary plexus) *n* = 10 (%) (7 children)	No eye abnormalities *n* = 20 (%) (8 children)	*P*-value
Disease duration (years)		10.3 ± 1.35	9.02 ± 1.28	0.039^*∗*^
Hb F (%) prior to study		6.67 ± 0.52	8.83 ± 3.91	0.192
Retics (%) prior to study		11.0 ± 2.37	8.87 ± 2.21	0.047^*∗*^

Exchange transfusion ^1^	One time (suspected stroke)	1/7 (14.3)	0/8 (0.0)	0.117
	One time (acute chest syndrome)	2/7 (28.6)	0/8 (0.0)	

Painful crisis	Frequent	5 (50.0)	2 (10.0)	0.040^*∗*^
	Infrequent	2 (20.0)	4 (20.0)	

Hydroxyurea therapy ^2^	Not compliant	4/7 (57.1)	1/8 (12.5)	0.046^*∗*^
	Compliant	1/7 (14.3)	6/8 (75.0)	

^1^Occurred in 3 children; all with eye abnormalities by OCT. ^2^Prescribed in 12 children; 7 with eye abnormalities and 5 without abnormalities by OCT.

## Data Availability

The data presented in this study can be obtained from the corresponding author upon request.
